# Effect of supplementation with brewer’s yeast hydrolysate on growth performance, nutrients digestibility, blood profiles and meat quality in growing to finishing pigs

**DOI:** 10.5713/ajas.18.0837

**Published:** 2019-02-14

**Authors:** Jian Ying Zhang, Jae Won Park, In Ho Kim

**Affiliations:** 1 Department of Animal Resource and Science, Dankook University, Cheonan 31116, Korea

**Keywords:** Brewer Yeast Hydrolysate, Growth Performance, Nutrients Digestibility, Blood Profile, Growing Pigs

## Abstract

**Objective:**

This study was aimed to investigate the effects of brewer’s yeast hydrolysate (YH) on growth performance, nutrients digestibility, blood profiles and meat quality of growing pigs.

**Methods:**

A total of 200 growing pigs ([Landrace×Yorkshire]×Duroc) (initial body weight, 25.31±1.29 kg) were allotted to 5 treatments as follow: CON, basic diet; and YH treatment, CON+0.05%, 0.1%, 0.5%, and 1.0% of YH, respectively.

**Results:**

On wk 11, 16 and overall phase, pigs fed YH diet showed a linear improvement in average daily body gain and gain/feed (p<0.05). The pigs that received YH linearly increased the digestibility of dry matter, nitrogen, and energy on wk 11 and 16. The concentration of serum urea nitrogen was linearly increased in YH treatments on wk 16. However, the carcass weight, back fat and lean muscle percentage of pigs receiving YH had no significant change. Besides, no difference was observed in creatinine and total protein in the blood among treatments.

**Conclusion:**

The pigs fed a graded YH diet had improved growth performance and nutrient digestibility, meanwhile, the YH increased the serum urea nitrogen in the growing pigs.

## INTRODUCTION

Over thousands of years, brewer yeast has been widely used for alcohol fermentation in the brewing process. Meanwhile, as eukaryote unicellular organisms, yeast has strong fermentation ability, which has a lower toxic potential with a high nutritional value [1–3]. Nowadays, yeast fermentation derivative products with bioactive properties including yeast cells and yeast extracts have been known to improve dietary palatability and fiber digestibility, prevent pathogen growth, produce antibacterial compounds and modulate the immune system [4–7].

Compared with yeast extracts, hydrolyzed yeast has a lower cost advantage by reducing production process without extraction. The methods of the manufacturing preparation of hydrolysates are divided into autolysis and enzymatic hydrolysis which were used to hydrolyze the yeast cells to release the nutrients. Results have indicated that enzymatic hydrolysis is the more effective method [8–10].

A downstream process of yeast extract production was developed from brewer’s yeast cells, including various processing conditions as clarification, debittering, and the Maillard reaction. Some studies followed a process of manufacture where the yeast cells were washed, centrifuged and then heat-inactivated before drum drying after fermentation [11]. However, in our process of production “yeast hydrolysate (YH)”, the process was shortened to treating the yeast cells with an enzyme mixture (mainly protease, cellulose, etc.) after liquid fermentation, then concentration of the resulting hydrolysate. This study was conducted to evaluate the effect of YH supplementation in diets on growth performance, nutrient digestibility, meat quality and blood profile in growing to finishing pigs.

## MATERIALS AND METHODS

### Animal care

The experiment was conducted at the swine experimental unit of Dankook University (Cheonan, Korea). The protocol for the current experiment was approved by the Animal Care and Use Committee of Dankook University.

### Source of brewer’s yeast hydrolysate

Brewer’s YH (*Saccharomyces cerevisiae*) was obtained from the Immunebio company (Eumseong, Korea). The Brewer’s yeast used in our study was provided by Plantinum Brewery Company (Seoul, Korea). Plantinum Brewery company mentioned that brewer’s yeast contains 4,240 kcal/kg digestible energy, 53.2% crude protein (CP), 1.8% crude fat (ether extract) (Method 920.39), 5.2% ash (Method 942.05) [12].

### The manufacture of a brewer’s yeast hydrolysate

Brewer’s yeast was inserted into the vessel with HCl (Cas No. 7647-001-0. Duksan corp., Seoul, Korea) and heated to the target temperature 105°C. When vessel temperature was 105°C, the sample was held for 4 h under the controlled temperature and cooled down to 45°C. After cooling, the samples were removed from the vessel, and passed through a mash filter (1.0×1.0 mm).

Brewer’s YH bioprocesses were by polysaccharide culture from the STR Biotech company, Ltd (Chuncheon, Korea). Brewer’s yeast and *L. edodes* fungal mycelia were isolated from the mushroom fruitbody and cultured on yeast mold agar (Difco Laboratory, Detroit, MI, USA) and potato dextrose agar medium (PDA, Difco Laboratory, USA). The brewer’s yeast with mycelia cultured on PDA media was inoculated in 50 mL of the liquid medium (Table 1). The culture experiments were commenced in 250 mL Erlenmeyer flasks at 28°C for 5 d in a rotary shaker (120 rpm) and that were then used to seed the main liquid culture. The main liquid medium contained black rice bran (20 g/L) and dried soybean powder (2 g/L). The medium was then treated with amylase and protease at 60°C for 60 min for enzymatic digestion of particulate materials containing carbohydrate and protein. Subsequently, the culture mass was adjusted to pH 6.0 with brewer’s YH, followed by sterilization in the autoclave. The experiment with the main liquid culture was started using a 5 L fermentor (working volume of 3 L) at 28°C and 150 rpm by inoculating with the inoculum (10%) of the pre-liquid cultured mycelia. After 7 days, the culture mass was ground in a colloid mill (model PUC60 Hankook Power Technology System, Seoul, Korea). The powder was treated with 0.1 M lactic acid for 60 min, followed by treatment with an enzyme mixture for cell wall lysis containing cellulase, hemicellulase, pectinase, glucanase, mannase, and arabinase at 50°C for 60 min.

### Experimental design, animals, and diets

A total of 200 crossbred growing pigs ([Landrace×Yorkshire] ×Duroc) with an average initial body weight of 25.31±1.29 kg were used in feeding trial for 16 weeks. Pigs were randomly allotted to 5 experiment treatments. Dietary treatments include: CON, basic diet; YH0.05, CON+0.05% YH; YH0.1, CON+0.10% YH; YH0.5, CON+0.50% YH; and YH1, CON+ 1.0% YH. There were 8 replicates per treatment with 5 pigs (three gilts and two barrows) per replicate. All diets were provided in mash form and formulated to meet or exceed the NRC [13] recommendations for all nutrients (Table 2) including growing pig phase (wk 1 to 6) and finishing pig phase (wk 6 to 16). All the pigs were housed in an environmentally controlled room with a slatted plastic floor. Throughout all the experimental period, each pen was equipped with a 1-sided self-feeder and a nipple drinker to allow the pig’s *ad libitum* access to feed and water.

### Sampling and measurements

#### Growth performance

Body weight and feed consumption were measured initially and wk 6, 11, and 16 to monitor the average daily gain (ADG), average daily feed intake (ADFI), and gain/feed (G/F) ratio.

#### Nutrients digestibility

Apparent total tract digestibility (ATTD) of dry matter (DM) and nitrogen (N) were determined by adding chromic oxide (0.2%) as an inert indicator in the diet. Pigs were fed diets mixed with chromic oxide one week before collecting samples at the last day of wk 6, 11, and 16. Fresh fecal grab samples collected from at least 2 pigs per pen by rectal massage were stored in a freezer at −20°C until analyzed. Before chemical analysis, the fecal samples were thawed and dried at 60°C for 72 h, after which they were finely ground to a size that could pass through a 1-mm screen. All feed and fecal samples were analyzed for DM and N following the procedures outlined by the AOAC [12]. Chromium was analyzed via UV absorption spectrophotometry (Shimadzu, UV-1201, Shimadzu, Kyoto, Japan) following the method described by Williams et al [14]. Energy (E) was determined by measuring heat of combustion in the samples, using a bomb calorimeter (Parr 6100; Parr Instrument Co., Moline, IL, USA). Nitrogen content was determined (Method 920.40) [12] and crude protein was calculated as N×6.25.

The ATTD was calculated using the following formula:

$$\text{ATTD}=1-[({\text{N}}_{\text{f}}\times {\text{C}}_{\text{d}})/({\text{N}}_{\text{d}}\times {\text{C}}_{\text{f}})]$$

Where N_f_ = nutrient concentration in faces (% DM), N_d_ = nutrient concentration in diet (% DM), C_d_ = chromium concentration in diet (% DM) and C_f_ = chromium dioxide concentration in faces (% DM).

#### Blood profiles and carcass grade X

At the end of the experiment (16 wk), blood samples were taken from each pig by Vacuum tube (Becton Dickinson Vacutainer Systems, Franklin Lakes, NJ, USA). Serum samples were isolated (centrifuged at 3,000×g for 15 min) 2 to 4 h after collection. An automatic blood analyzer (HITACHI 747, Kyoto, Japan) was used analyze blood urea nitrogen (BUN), total protein and creatinine in the serum samples.

At the end of the experiment (16 wk) the pigs were slaughtered in a slaughterhouse and a report on carcass grade was received from the web site of Korea Institute for Animal Products Quality Evaluation.

#### Statistical processing

The data were analyzed using the general linear model procedure of SAS (SAS Institute, Inc., Cary, NC, USA, 1996) as a randomized complete block design. Pen served as the experimental unit. Orthogonal comparisons were conducted using polynomial regression to measure the linear, quadratic, and cubic effects of increasing the supplementation YH. Results were expressed as the least squares means and standard error of mean. Probability values less than 0.05 were considered significant.

## RESULTS

### Growth performance

The effects of YH on growth performance are shown in Table 3. On wk 11, 16, and overall period, the ADG linearly increased for pigs fed YH, meanwhile, the ADG of pigs cubically increased and G/F was linearly improved on wk 6 (p<0.05). Moreover, the G/F of pigs fed YH also linearly increased since they consumed YH at every stage of the experiment. There was no significant effect of increased content of ADFI during experiment phase.

### Nutrient digestibility

The results of nutrient digestibility are shown in Table 4. On wk 11, only digestibility of DM (p = 0.002) and N (p = 0.030) were linearly increased in the response of increasing dietary concentrations of supplemental YH. On wk 16, there was a linear increased in DM (p = 0.004), N (p = 0.030), and E (p = 0.021) in the response to increasing dietary concentrations of supplemental YH. However, on wk 6, there were no significant effects on nutrient digestibility among treatments.

### Meat quality

The results of carcass grading are shown in Table 5. There was no significant difference in meat quality among the treatments.

### Blood profile

Table 6 shows results of blood profile from growing-finishing pigs. There was no significant difference among the treatments in creatinine and total protein. However, the BUN of pigs linearly increased as the inclusion rate of YH increased (p = 0.049).

## DISCUSSION

### Growth performance

The yeast cell and extract has used in the livestock industry for many decades. Many previous studies demonstrated that yeast cells have a positive effect on growth performance of pigs. Li et al [15] reported that the live yeast (*Saccharomyces cerevisiae*) increased quadratically body weight gain and feed intake during a starter experiment with weaning pigs. Meanwhile, Håkenåsen [11] showed that the ADG was linearly increased by increasing grade of yeast (*Candida utilis*) in weanling piglets. Those observations are consistent with the finding of Sun et al [16] who indicated that the ADG and ADFI were increased in nursery pigs fed a diet with added 2 g/kg yeast cells.

In the present study, the ADG and G/F were linearly increased in the overall period by diet with added graded YH (from 0.05% to 1%). Few papers about YH used in the diet were found which lead us to consider other yeast derivatives. Our results are consistent with Dvorak and Jacques [17] and Li et al [18] that a diet with added yeast extract has a positive effect on the body weight gain and growth efficiency in the pigs. Meanwhile, Zhao et al [19] found that the ADG and ADFI were increased in the pigs fed a nursery diet containing yeast extract. The reason ADFI was increased may be due to improving palatability in the dietary after adding yeast. A similar viewpoint that pigs receiving diets with higher levels of yeast had improved the feed intake [3,11]. Furthermore, some researchers thought that the yeast cells which were treated with multiple enzymes had a better flavor profile since the enzymes strongly affected the degree of hydrolysis and protein compositional characteristics [20].

According to Kemp and Kiser [21] glucan as the main component of the yeast cells, which has significant influence on growth performance of animals. The probable reason is that yeast cells produced and released various proteolytic, glycolytic, or lipolytic enzymes to digest organic matter, or absorbed amino acids and monosaccharides which has the effect of suppressing bacteria, thereby increasing growth performance. A similar explanation is that growth performance was improved by assimilation of nutrients that are directly used for growth and because of β-glucan inhibits the production of cytokines, which can be produced by pathogenic microbial infections in the animal [22]. In addition, some papers found that a diet with added yeast had a significant influence on the bone growth, for example, tibial bone, bonefemur bone growths and growth plate (proximal epiphysis) in the rat [23]. The mechanism of the positive effects on animals produced by a diet with relevant yeast needs to be researched.

### Digestibility nutrients

As described above, after treated by enzyme mixture, the mixture of yeast cells and culture increased not only feed intake but also digestibility of nutrients. As many studies have demonstrated the pig diet with added protease, cellulose or enzyme mixture could apparently enhance the digestibility of crude protein, energy, and dry mater, thereby improving the growth performance of pigs [24–26]. Those results are consistent with our research that the digestibility of DM and N were increased at wk 11 and the digestibility of E was increased at wk 16 by diet concentration of supplemental YH which contributed to the growth performance in pigs.

Our results are supported by Keimer et al [6] that the digestibility of CP and E tended to increase in 1.0% hydrolyzed yeast group. We speculate that the healthy morphology of the intestine was improved by diet with added YH. Håkenåsen [11] researched that fecal DM was linearly increased after adding levels of yeast, meanwhile, Keimer et al [6] showed that weaned pigs had significantly increased villus height, villus/crypt ratio in jejunum, and reduced crypt depth in the colon when fed a diet with added 1% hydrolyzed yeast. Carlson [27] has more detailed observations that after being fed a yeast diet, nursery pigs had shorter crypt depths which means that fewer cells were migrating to the villus to ad digestion and absorption. However, different results have reported that short-term supplementation with dietary yeast [15,27] in the diet of weaned pigs had no influence on the ileal DM, CP, and crude fiber digestibility.

It is a hypothesized that the β-glucan may positively effect digestion and absorption rate by increasing the immune system and maintaining health in the state of infectious diseases. Hiss et al [22] reported that the nutrient utilization rate (digestibility of DM, E, CP, calcium, and phosphorous) of young piglets fed β-glucan was significantly improved. But it needs further research into how β-glucan directly affects the digestibility of nutrients.

### Meat quality

That dietary supplementing with yeast could favorably improve the quality of edible meat from broilers has been proved by many trials. For example, edible meats from broiler chicks fed a diet containing enriched yeast (*Saccharomyces cerevisiae*) exhibited increased tenderness and increased water holding capacity [28,29]. Some results showed that dietary 0.3% yeast supplementation improved the antioxidant status in muscle. Meanwhile, increased levels of yeast decreased the drip loss and the concentration of thiobarbituric acid reactive substances in the muscle and meat [30]. Another point is that the addition of yeast to the diet improves the quality of meat by decreasing fatty acids. In a study of diet added 0.5% yeast extract to the broiler, the oxidative fat was decreased [30]. Onifade et al [31] researched that a diet supplement of *Saccharomyces cerevisiae* to broilers decreased abdominal fat mass. We searched the literature and found that the probable reason yeast had significant influenced on the birds’ meat was due to the yeast improving the oxidative stability of meat.

However, the data was limited for the effect of yeast cells or yeast metabolic products on meat quality in pigs. In the present paper, carcass weight, back fat and lean muscle percentage of pigs were not significantly different. As opposed to a paper that carcass weight and breast muscles were increased by a diet containing *Saccharomyces cerevisiae* yeast cells [32]. Therefore, further research is required to investigate the effect of yeast on the quality of meat in pigs.

### Blood profile

The response of blood profile in the diet supplemented with graded of YH is shown in Table 6 where serum urea nitrogen was increased, while creatinine and total protein were not influenced in the pigs. We speculated that the diet promoted N absorption after added YH and for this is reason the digestibility of N was increased in our experiment. However, studies about blood profiles effects of brewer’s yeast are limited. A similar result that the plasma of serum urea nitrogen was increased by diet contained hydrolyzed yeast in weaned pigs has been reported [33]. It has been discovered that diet with added yeast extract could decrease the content of cholesterol in the blood. One study was reported that β-glucan from Saccharomyces cerevisiae fed to high cholesterol group of men decreased total cholesterol in the blood [34].

Otherwise, we searched the literature for the influence on other blood profiles in animals fed yeast. We found that a diet supplement of YH could help animals increase growth hormones (GHs) and immune responses. For example, Kim et al [23] demonstrated that the diet with a maximum of 1.0% of yeast enhanced GH in the rats. That GH was increased by adding yeast in the diet is a probable reason why the pigs could improve the growth performance after fed a yeast diet in the present experiment. Elsewhere, some papers directly demonstrated that yeast metabolites could promote improving immune responses. Waititu et al [33] found that yeast added multi-enzyme mixture could reduce ileal interferon-γ and interleukin-10 expression after challenged *Escherichia coli* lipopolysaccharide in weaned pigs. Jeney and Anderson [35] demonstrated that yeast extract promoted specific and non-specific immune responses by enhancing the acid phosphates activity of peritoneal macrophages, neutrophil activity and produce oxidative radicals. On blood profiles, there are needs to further study various effects of hydrolyzed brewer’s yeast.

## CONCLUSION

The effect of increasing supplementation with brewer’s YH was to improve the growth performance with body weight and feed efficiency, besides, the apparent digestibility of nutrients in some phases was significantly increased, and the content of blood urea nitrogen was improved in the growing pigs. However, there was no significant effect on creatinine, total protein and carcass grade in growing to finishing pig.

## Figures and Tables

**Table 1:** Composition of liquid media

Ingredient	Amount (%)
Glucose	2.0
Yeast extract	0.5
Soy peptone	0.5
KH_2_PO_4_	0.2
MgSO_4_	0.05
FeSO_4_	0.002
Distilled water	96.748

**Table 2:** Ingredient composition of experimental diets as-fed basis

Items	Growing phase	Finishing phase
Ingredient (%)		
Corn	51.47	49.39
Wheat	17.08	20.96
Soybean meal	12.08	10.40
Palm kernel meal	2.18	2.24
DDGS	5.00	7.00
Meat meal	4.76	2.34
Animal fat	3.00	3.00
Molasses	3.00	3.00
Calcium monophosphate	0.18	0.24
Limestone	0.68	0.87
Choline chloride50	0.12	0.10
Salt	0.25	0.26
Vitamin premix1)	0.10	0.10
Minerals premix2)	0.10	0.10
Calculated composition		
Crude protein (%)	16.50	15.00
Fat (%)	5.70	5.60
Fiber (%)	3.70	3.90
Calcium (%)	0.66	0.59
Phosphorus (%)	0.57	0.54
Metabolizable energy (kcal/kg)3)	3,360	3,348
Lysine (%)	0.98	0.88
Methionine (%)	0.36	0.32

DDGS, dry distillers grains with solubles.

1) Provided per kilogram of complete diet: 1.3 mg vitamin A (retinol); 0.022 mg vitamin D_3_ (cholecalciferol); 45 mg vitamin E (tocotrienol); 4.2 mg vitamin K_3_ (menodione); 24.6 mg vitamin B_5_ (d-Ca-pantothenate); 8.6 mg vitamin B_2_ (riboflavin); 0.04 mg vitamin B_12_ (cobalamins).

2) Provided per kilogram of complete diet: 15 mg Cu; 80 mg Fe; 56 mg Zn; 73 mg Mn; 0.3 mg I; 0.5 mg Co; 0.4 mg Se.

3) Metabolizable energy was calculated by ingredient metabolizable energy from NRC [13].

**Table 3:** Effect of brewer’s yeast hydrolysate supplementation on growth performance in growing - finishing pigs

Items	CON1)	YH0.051)	YH0.11)	YH0.51)	YH1.01)	SEM2)	p-value3)		

							Linear	Quadratic	Cubic
Body weight (kg)									
Initial	25.33	25.38	25.23	25.38	25.2	0.07	0.15	0.51	0.89
wk 6	52.06	54.11	52.59	52.62	52.68	0.43	0.99	0.52	0.03
wk 11	77.83	79.6	79.72	80.04	81.78	0.70	0.01	0.69	0.76
wk 16	106.54	108.67	109.4	109.48	112.29	0.87	0.01	0.82	0.76
Wk 6									
Average daily gain (g)	637	684	652	648	654	10	0.80	0.54	0.02
Average daily feed intake (g)	1,610	1,617	1,643	1,547	1,521	41	0.05	0.54	0.28
Gain/feed	0.396	0.423	0.397	0.420	0.431	0.008	0.01	0.71	0.70
Wk 11									
Average daily gain (g)	736	771	775	782	790	13	0.01	0.94	0.15
Average daily feed intake (g)	2,103	2,111	2,173	2,141	2,135	24	0.19	0.30	0.55
Gain/feed	0.350	0.365	0.357	0.365	0.370	0.006	0.01	0.42	0.24
Wk 16									
Average daily gain (g)	820	831	848	841	872	15	0.01	0.89	0.94
Average daily feed intake (g)	2,736	2,764	2,829	2,766	2,800	43	0.44	0.39	0.69
Gain/feed	0.300	0.300	0.300	0.304	0.311	0.003	0.01	0.15	0.71
Overall									
Average daily gain (g)	725	744	752	753	776	8	0.01	0.78	0.73
Average daily feed intake (g)	1,517	1,499	1,560	1,502	1,546	20	0.65	0.49	0.71
Gain/feed	0.478	0.496	0.482	0.501	0.502	0.005	0.01	0.56	0.39

YH, yeast hydrolysate; SEM, standard error of means.

1) CON, basic diet; YH0.05, CON+0.05% YH; YH0.1, CON+0.10% YH; YH0.5, CON+0.50% YH; YH1.0, CON+1.0% YH.

2) Each mean represents 8 observations per treatment.

3) p<0.05 was considered significant difference.

**Table 4:** Effect of brewer’s yeast hydrolysate supplementation on the nutrient digestibility in growing - finishing pigs

Items (%)	CON1)	YH0.051)	YH0.11)	YH0.51)	YH1.01)	SEM2)	p-value3)		

							Linear	Quadratic	Cubic
Wk 6									
Dry matter	79.89	80.25	80.14	80.20	80.93	0.75	0.611	0.708	0.703
Nitrogen	78.33	78.35	78.10	78.25	79.11	1.04	0.704	0.880	0.716
Energy	80.84	80.63	80.74	80.64	80.58	0.74	0.913	0.854	0.977
Wk 11									
Dry matter	73.86	74.29	74.19	75.56	76.20	0.77	0.001	0.602	0.690
Nitrogen	74.23	75.58	74.73	76.52	75.55	0.96	0.034	0.765	0.456
Energy	76.31	75.91	77.44	76.95	77.61	0.74	0.089	0.788	0.691
Wk 16									
Dry matter	70.41	71.42	71.20	71.56	73.16	0.74	0.003	0.791	0.979
Nitrogen	69.30	70.63	70.71	70.46	72.32	0.90	0.025	0.803	0.903
Energy	70.25	71.03	70.80	70.79	72.28	0.72	0.022	0.604	0.841

YH, yeast hydrolysate; SEM, standard error of means.

1) CON, basic diet; YH0.05, CON+0.05% YH; YH0.1, CON+0.10% YH; YH0.5, CON+0.50% YH; YH1.0, CON+1.0% YH.

2) Each mean represents 8 observations per treatment.

3) p<0.05 was considered significant difference.

**Table 5:** Effect of brewer’s yeast hydrolysate supplementation on carcass grade in growing - finishing pigs

Items	CON1)	YH0.051)	YH0.11)	YH0.51)	YH1.01)	SEM2)	p-value3)		

							Linear	Quadratic	Cubic
Carcass weight (kg)	88.01	87.49	87.33	88.52	87.91	0.82	0.242	0.343	0.856
Back fat (mm)	19.59	19.74	20.23	20.28	20.47	1.07	0.861	0.314	0.488
Lean muscle percentage (%)	54.82	54.15	55.13	55.61	55.02	0.39	0.069	0.839	0.434

YH, yeast hydrolysate; SEM, standard error of means.

1) CON, basic diet; YH0.05, CON+0.05% YH; YH0.1, CON+0.10% YH; YH0.5, CON+0.50% YH; YH1.0, CON+1.0% YH.

2) Each mean represents 8 observations per treatment.

3) p<0.05 was considered significant difference.

**Table 6:** Effect of brewer’s yeast hydrolysate supplementation on blood profile in growing - finishing pigs

Items (mg/dL)	CON1)	YH0.051)	YH0.11)	YH0.51)	YH1.01)	SEM2)	p-value3)		

							Linear	Quadratic	Cubic
Serum urea nitrogen	10.50	10.50	10.25	12.25	11.25	0.67	0.052	0.901	0.533
Creatinine	1.05	1.07	1.10	1.07	0.97	0.07	0.556	0.671	0.415
Total protein	6.73	6.60	6.69	6.67	6.76	0.34	0.858	0.873	0.817

YH, yeast hydrolysate; SEM, standard error of means.

1) CON, basic diet; YH0.05, CON+0.05% YH; YH0.1, CON+0.10% YH; YH0.5, CON+0.50% YH; YH1.0, CON+1.0% YH.

2) Each mean represents 8 observations per treatment.

3) p<0.05 was considered significant difference.

## References

[1] Kuhad RC, Singh A, Tripathi KK, Saxena RK, Eriksson KEL (1997). Microorganisms as an alternative source of protein. Nutr Rev.

[2] Vieira E, Moura C, Almeida T (2012). Influence of serial repitching on beer polypeptide profiles. J Am Soc Brew Chem.

[3] Pancrazio G, Cunha SC, Pinho PGD (2016). Spent brewer’s yeast extract as an ingredient in cooked hams. Meat Sci.

[4] Wongsasak U, Chaijamrus S, Kumkhong S, Boonanuntanasarn S (2015). Effects of dietary supplementation with β-glucan and synbiotics on immune gene expression and immune parameters under ammonia stress in pacific white shrimp. Aquaculture.

[5] Lima LM, Silva JW, Ogoshi RCS (2016). Evaluation of raw yeast extract (*Saccharomyces cerevisiae*) as an ingredient, additive or palatability agent in wet diet for cats. Int J Biol.

[6] Keimer B, Kröger S, Röhe I, Pieper R, Simon A, Zentek J (2018). Influence of differently processed yeast (*Kluyveromyces fragilis*) on feed intake and gut physiology in weaned pigs. J Anim Sci.

[7] Shurson GC (2018). Yeast and yeast derivatives in feed additives and ingredients: sources, characteristics, animal responses, and quantification methods. Anim Feed Sci Technol.

[8] Nagodawithana T (1992). Yeast-derived flavors and flavor enhancers and their probable mode of action. Food Technol.

[9] Jiang M, Chen KQ, Liu Z, Wei P, Ying H, Chang H (2010). Succinic acid production by *Actinobacillus succinogenes* using spent brewer’s yeast hydrolysate as a nitrogen source. Appl Biochem Biotechnol.

[10] Podpora B, Świderski F, Sadowska A, Piotrowska A, Rakowska R (2015). Spent brewer’s yeast autolysates as a new and valuable component of functional food and dietary supplements. J Food Process Technol.

[11] Håkenåsen IM (2017). Feed intake, nutrient digestibility, growth performance and general health of piglets fed increasing levels of yeast [master’s thesis].

[12] AOAC (2012). Official methods of analysis of the Association of Official Analytical Chemists International.

[13] NRC (2012). Nutrient requirements of swine.

[14] Williams CH, David DJ, Iismaa O (1962). The determination of chromic oxide in faeces samples by atomic absorption spectrophotometry. J Agric Sci.

[15] Li J, Li D, Gong L, Ma Y, He Y, Zhai H (2006). Effects of live yeast on the performance, nutrient digestibility, gastrointestinal microbiota and concentration of volatile fatty acids in weanling pigs. Arch Anim Nutr.

[16] Sun Y, Park I, Guo J, Weaver AC, Kim SW (2015). Impacts of low level aflatoxin in feed and the use of modified yeast cell wall extract on growth and health of nursery pigs. Anim Nutr.

[17] Dvorak R, Jacques KA (1998). Mannanoligosaccharide, fructooligosaccharide, and carbadox for pigs days 0–21 post-weaning. J Anim Sci.

[18] Li J, Li DF, Xing JJ, Cheng ZB, Lai CH (2006). Effects of β-glucan extracted from sacharomyces cerevisiae on growth performance and immunological and somatotropic responses of pigs challenged with *Escherichia coli* lipopolysaccharide. J Anim Sci.

[19] Zhao PY, Jung JH, Kim IH (2012). Effect of mannan oligosaccharides and fructan on growth performance, nutrient digestibility, blood profile, and diarrhea Score in Weanling Pigs. J Anim Sci.

[20] Chae HJ, Joo H, In MJ (2001). Utilization of brewer’s yeast cells for the production of food-grade yeast extract. Part 1: effects of different enzymatic treatments on solid and protein recovery and flavor characteristics. Bioresour Technol.

[21] Kemp G, Kiser J (1970). Microbial resistance and public health aspects of use of medicated feeds. J Anim Sci.

[22] Hiss S, Sauerwein H (2003). Influence of dietary β-glucan on growth performance, lymphocyte proliferation, specific immune response and haptoglobin plasma concentrations in pigs. J Anim Physiol Anim Nutr.

[23] Kim JM, Kim SY, Jung EY, Bae SH, Suh HJ (2009). Yeast hydrolysate induces longitudinal bone growth and growth hormone release in rats. Phytother Res.

[24] Varel VH, Robinson IM, Jung HJ (1987). Influence of dietary fiber on xylanolytic and cellulolytic bacteria of adult pigs. Appl Environ Microbiol.

[25] Caine WR, Sauer WC, Tamminga S, Verstegen MWA, Schulze H (1997). Apparent ileal digestibilities of amino acids in newly weaned pigs fed diets with protease-treated soybean meal. J Anim Sci.

[26] Yin YL, Baidoo SK, Jin LZ, Liu YG, Schulze H, Simmins PH (2001). The effect of different carbohydrase and protease supplementation on apparent (ileal and overall) digestibility of nutrients of five hulless barley varieties in young pigs. Livest Prod Sci.

[27] Carlson MS (2005). Effects of yeast extract versus animal plasma in weanling pig diets on growth performance and intestinal morphology. J Swine Health Prod.

[28] Bonomi AB, Bonomi M, Quarantelli A (1999). Organic chromium in the feeding of the broilers. Rivista Di Scienza Dellalimentazione.

[29] Lee HS, Noh DO, Suh HJ (2011). Promotion effects of yeast hydrolysates and a mixture of safflower seed and gasiogapi extract on longitudinal bone, proximal epiphysis, and growth hormone in rats. Prev Nutr Food Sci.

[30] Li JG, Zhou JC, Zhao H (2011). Enhanced water-holding capacity of meat was associated with increased sepw1 gene expression in pigs fed selenium-enriched yeast. Meat Sci.

[31] Onifade AA, Odunsi AA, Babatunde GM, Olorede BR, Muma E (1999). Comparison of the supplemental effects of *Saccharomyces cerevisiae* and antibiotics in low-protein and high-fibre diets fed to broiler chickens. Arch Tierernaehr.

[32] Zhang AW, Lee BD, Lee SK (2005). Effects of yeast (*Saccharomyces cerevisiae*) cell components on growth performance, meat quality, and ileal mucosa development of broiler chicks. Poult Sci.

[33] Waititu SM, Heo JM, Patterson R, Nyachoti CM (2016). Dietary yeast-based nucleotides as an alternative to in-feed antibiotics in promoting growth performance and nutrient utilization in weaned pigs. Can J Anim Sci.

[34] Nicolosi R, Bell SJ, Bistrian BR, Greenberg I, Forse RA, Blackburn GL (1999). Plasma lipid changes after supplementation with β-glucan fiber from yeast. Am J Clin Nutr.

[35] Jeney G, Anderson DP (1993). Glucan injection or bath exposure given alone or in combination with a bacterin enhance the non-specific defence mechanisms in rainbow trout (*Oncorhynchus mykiss*). Aquaculture.

